# The tetrameric MotA complex as the core of the flagellar motor stator from hyperthermophilic bacterium

**DOI:** 10.1038/srep31526

**Published:** 2016-08-17

**Authors:** Norihiro Takekawa, Naoya Terahara, Takayuki Kato, Mizuki Gohara, Kouta Mayanagi, Atsushi Hijikata, Yasuhiro Onoue, Seiji Kojima, Tsuyoshi Shirai, Keiichi Namba, Michio Homma

**Affiliations:** 1 Division of Biological Science, Graduate School of Science, Nagoya University, Chikusa-ku, Nagoya 464-8602, Japan; 2 Graduate School of Frontier Biosciences, Osaka University, 1-3 Yamadaoka, Suita, Osaka 565-0871, Japan; 3 Medical Institute of Bioregulation, Kyushu University, Higashi-ku, Fukuoka 812-8581, Japan; 4 JST, PRESTO, Fukuoka 812-8582, Japan; 5 Department of Bioscience, Nagahama Institute of BioScience and Technology, 1266 Tamura, Nagahama, 526-0829, Japan

## Abstract

Rotation of bacterial flagellar motor is driven by the interaction between the stator and rotor, and the driving energy is supplied by ion influx through the stator channel. The stator is composed of the MotA and MotB proteins, which form a hetero-hexameric complex with a stoichiometry of four MotA and two MotB molecules. MotA and MotB are four- and single-transmembrane proteins, respectively. To generate torque, the MotA/MotB stator unit changes its conformation in response to the ion influx, and interacts with the rotor protein FliG. Here, we overproduced and purified MotA of the hyperthermophilic bacterium *Aquifex aeolicus*. A chemical crosslinking experiment revealed that MotA formed a multimeric complex, most likely a tetramer. The three-dimensional structure of the purified MotA, reconstructed by electron microscopy single particle imaging, consisted of a slightly elongated globular domain and a pair of arch-like domains with spiky projections, likely to correspond to the transmembrane and cytoplasmic domains, respectively. We show that MotA molecules can form a stable tetrameric complex without MotB, and for the first time, demonstrate the cytoplasmic structure of the stator.

Various motor proteins are essential for different biological activities such as cell locomotion, cell morphogenesis, metabolism, and material transport. Motor proteins convert various types of energy, such as ATP hydrolysis or electrochemical potential, into mechanical force for directional motion. Motor proteins are divided into two types: linear motors, such as myosin-actin and kinesin/dynein microtubule motors; and rotary motors, such as ATP synthase and bacterial flagellar motor. The bacterial flagellar motor is composed of about 20 different proteins, and drives the rotation of the helical flagellar filament as a propeller for the locomotion of the bacterial cell. Torque of the flagellar motor is generated by the interaction between two components: the rotor and the stator. The rotor is composed of several rings with a central shaft. The stator is the energy-converting unit that works as an ion channel and interacts with the rotor. It is believed that the ion influx through the channel induces a conformational change in the stator, allowing its interaction with the rotor to generate torque^1^,^2^.

The stator is composed of two transmembrane proteins, MotA and MotB (Fig. S1). MotA has four transmembrane helices and a large cytoplasmic domain between the second and third transmembrane helices^3^. This cytoplasmic domain is thought to interact with a rotor protein, FliG^4^. MotB has a single transmembrane helix and a large periplasmic C-terminal domain, composed of a plug region for the channel, a peptidoglycan-binding region, and a flexible linker that connects these two parts^5^,^6^,^7^. MotA and MotB form a MotA_4_MotB_2_ hetero-hexameric complex, containing an ion channel formed by the third and fourth transmembrane regions of MotA and a transmembrane region of MotB^8^,^9^,^10^. Previous studies on characterizing the stator were performed using purified MotA/MotB or its homologous proteins. PomA and PomB are homologs of MotA and MotB, respectively, in the sodium-driven flagellar motor of *Vibrio alginolyticus*. Purified PomA and PomB formed a PomA_4_PomB_2_ hexameric complex, whereas PomA alone formed a PomA_2_ homodimer when solubilized in detergent octyl β-D-glucopyranoside (OG)^11^,^12^. The MotA/MotB purified from the proton-driven motor of *Escherichia coli* also formed a MotA_4_MotB_2_ complex when solubilized in detergent n-dodecylphosphocholine (DPC)^10^. The purified PomA/PomB complex is functional as an ion channel when reconstituted into membrane vesicles^12^. The topology and arrangement of the transmembrane regions of the MotA/MotB complex were analyzed by disulfide crosslinking experiments^8^,^9^ and molecular dynamics simulation^13^. The crystal structures of the periplasmic peptidoglycan-binding domains of MotB and PomB show folds similar to those of other peptidoglycan-binding proteins^14^,^15^,^16^,^17^,^18^. The three-dimensional (3D) structure of the purified PomA/PomB complex, solubilized in detergent Cymal-5 and determined by electron microscopy (EM) single particle image analysis, revealed a globular shape with two periplasmic arms^19^.

The crystal structure of full length FliG from hyperthermophilic *Aquifex aeolicus* has been described previously^20^. FliG is thought to form a ring of approximately 30-fold rotational symmetry, and to directly interact with MotA to generate torque^4^,^20^,^21^,^22^,^23^. Recently, we reported that the MotA/MotB complex of *A. aeolicus* can function in the motor of *E. coli* after two modifications: the replacement of the periplasmic region of MotB with that of *E. coli;* and the introduction of a point mutation at the C-terminal region of MotA (A225D)^24^. In the present study, we characterized the purified native MotA protein of *A. aeolicus*. We screened conditions to optimize the purification method, and characterized the multimeric complex formation and structure by biochemical methods and EM image analysis.

## Results

### Screening of stator proteins and detergents

First, we cloned the genes encoding the stator proteins of the flagellar motor from various bacterial strains to identify highly expressing and stable stator proteins. The *motA* and *motB* genes or their orthologs were cloned from a marine bacterium, *V. alginolyticus*; three piezophilic bacteria, *Photobacterium profundum*, *Shewanella benthica*, and *Shewanella violacea*; and two thermophilic bacteria, *Thermotoga maritima* and *A. aeolicus*, into two plasmids, pColdI and pBAD33. When *motA*/*motB* were cloned into pColdI, a six histidine (His_6_) tag was fused to the N-terminus of the produced MotA protein. *E. coli* cells were transformed with the pColdI-based plasmid, and the production of MotA protein was tested by sodium dodecylsulfate-polyacrylamide gel electrophoresis (SDS-PAGE) and immunoblotting using an anti-His antibody (Figs S2A and S2B). We also tested the function of the stator proteins expressed from the pBAD33-based plasmids in *E. coli*. We previously reported that the stator composed of *A. aeolicus* MotA and chimeric MotB functioned in *E. coli* flagellar motor^24^, and here we found that some of the stator from other species were also functional in the different hosts (Fig. S3 and Table S2). From the SDS-PAGE experiment, we found that MotA^Aa^ (MotA from *A. aeolicus*) showed the most stable and clear band (Fig. S2C) and therefore, decided to use this protein in this study. The whole-cell sample harboring the plasmid containing *motA*^*Aa*^/*motB*_*1*_^*Aa*^ was disrupted by sonication and separated into soluble cytoplasmic and insoluble membrane fractions by ultracentrifugation. Then, the membrane fraction was solubilized by detergent Cymal-5, and the MotA^Aa^ and MotB^Aa^ proteins were affinity-purified using a hexahistidine tag (Fig. S2C). Although both *motA*^*Aa*^ and *motB*_*1*_^*Aa*^ were cloned into a plasmid as operons, only MotA^Aa^ was purified (Fig. S2D). Therefore, we used a plasmid expressing only *motA*^*Aa*^ (pColdI-*motA*^*Aa*^) for the following experiments. *A. aeolicus* is known to have another *motB*^*Aa*^ gene, *motB*_*2*_^*Aa*^, immediately downstream of *motB*_*1*_^*Aa*^. We also cloned the *motB*_*2*_^*Aa*^ gene alone and overproduced it in *E. coli* cells, but MotB_2_^Aa^ was not soluble by Cymal-5 (Fig. S2E).

**Figure 1:**
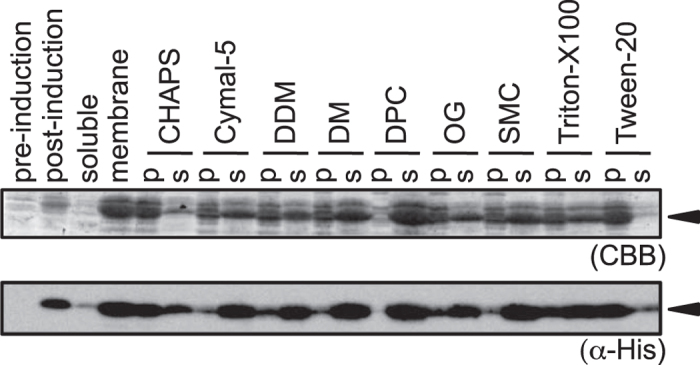
Results for solubilization efficiency of MotA protein from membrane fractions in 1% (w/v) of nine different detergents. Soluble and insoluble fractions were separated by ultracentrifugation. p, pellet; s, supernatant.

To screen the most effective detergents for solubilization of the MotA^Aa^ protein, the membrane fraction was treated with various detergents at a concentration of 1% (w/v), and the soluble and insoluble fractions were separated by ultracentrifugation as the supernatant and pellet fractions, respectively (Fig. 1). Among the nine detergents tested in this study, DPC, Cymal-5, DDM, DM and SMC were effective for the solubilization of MotA^Aa^. Consequently, we decided to use Cymal-5, DDM and DM for the purification of the MotA^Aa^ protein in this study.

### Purification of MotA

**Figure 2:**
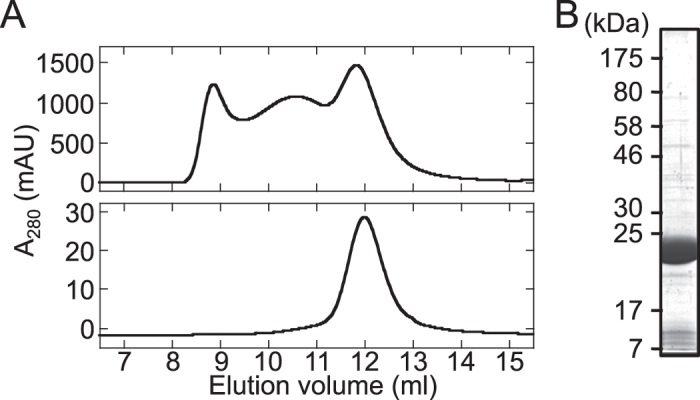
Purification of MotA. (**A**) Elution profile of purified MotA protein with 0.1% (w/v) DDM by Superdex 200 Increase 10/300 GL gel filtration chromatography. The upper panel shows the chromatogram obtained using samples collected just after purification by His-affinity beads, and the lower panel shows a chromatogram obtained for samples from the fraction collected during the first run, whose elution volume was approximately 12 mL. (**B**) Results for gel electrophoresis obtained for the MotA protein purified in (**A**).

The MotA^Aa^ (hereafter, simply described as MotA) protein was solubilized in DDM as described above and purified using histidine-affinity beads and gel filtration chromatography. By gel filtration chromatography, three peaks were obtained for the MotA protein. The apparent sizes of the complexes in these three fractions were estimated from their elution peak positions: ca. 230 kDa, ca. 420 kDa, and more than ca. 600 kDa (beyond the void volume) (Fig. 2A, top). When the fraction containing the 230-kDa complex was separated by gel filtration again, a single peak was detected with an estimated size of ca. 210 kDa (Fig. 2A, bottom). Coomassie brilliant blue (CBB) staining and immunoblotting analysis using anti-His antibody showed that this stable peak of ca. 210 kDa contained highly purified MotA protein (Fig. 2B). This size of 210 kDa is significantly larger than 28 kDa, the molecular mass of MotA with N-terminal His tag, suggesting that the purified MotA formed a stable multimeric complex.

### MotA alone can form at least a tetramer

**Figure 3:**
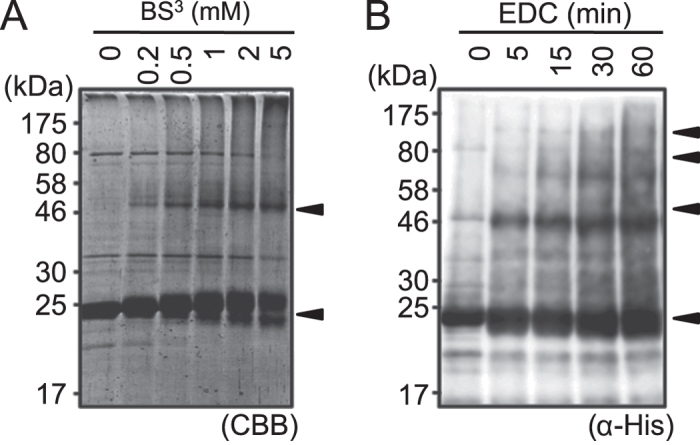
Crosslinking products of MotA. The purified MotA proteins were reacted with various concentrations of BS^3^ for 5 min (**A**) or with 20 mM EDC (**B**) for increasing lengths of time. The concentrations of BS^3^ (mM) or reaction times with EDC (min) are indicated on top of the panel.

To estimate the stoichiometry of MotA in the purified complex, we performed a chemical crosslinking experiment (Fig. 3). Purified protein was crosslinked by either BS^3^, a homo-bifunctional crosslinker that forms a bridge between amino groups, or EDC, a hetero-bifunctional crosslinker that forms a bridge between carboxyl and amino groups. Crosslinked products were analyzed by SDS-PAGE and immunoblotting. Without the chemical crosslink treatments, there was a clear single band with a molecular mass of ca. 25 kDa, which is in good agreement with that of monomeric His-MotA (28 kDa) (Figs 2B and 3A). Upon increasing the BS^3^ concentration, a band with a molecular mass of ca. 50 kDa appeared (Fig. 3A), probably corresponding to the MotA dimer. A similar result was observed with EDC, but with additional bands at ca. 75 kDa and ca. 100 kDa, corresponding to the MotA trimer and tetramer (Fig. 3B). Thus, purified MotA was found to form a multimeric complex composed of four or more MotA molecules.

### Surface accessibility of amino acid residues in MotA

**Figure 4:**
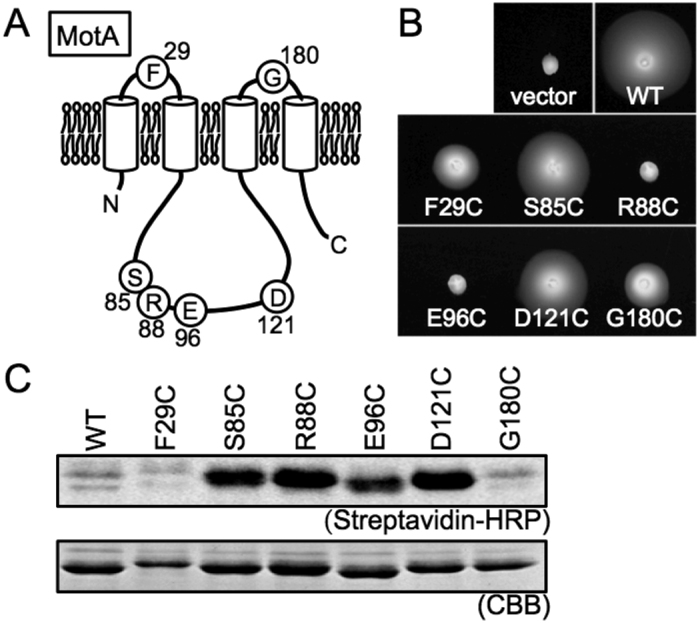
Cys substitutions of MotA. (**A**) Schematic representation of MotA displaying the mutated residues. (**B**) Motility assay of *E. coli* cells producing MotA (with or without mutation) and chimeric MotB of *A. aeolicus* in soft-agar plates. Overnight cultures were spotted on TB-0.25% agar plate containing 0.02% arabinose and 0.5 mM IPTG, and incubated at 30 °C for 26 h. (**C**) Surface accessibility of mutated residues in MotA. Cys residues in mutant MotA protein were labeled with biotin-maleimide and detected with streptavidin-conjugated HRP.

To examine the surface accessibility of amino acid residues of the MotA protein, cysteine substitutions were introduced into MotA (Fig. 4). We examined the motor function of the cysteine substitution mutants in *E. coli* using a hybrid mutant motor consisting of *A. aeolicus* MotA and chimeric MotB composed of *A. aeolicus* MotB and *E. coli* MotB^24^. The cysteine substitution mutants were functional except for R88C and E96C, which are mutations of conserved charged residues important for the interaction with the rotor protein FliG (Fig. 4B)^4^,^25^. The mutant MotA proteins were purified and treated with biotin maleimide, and the bound biotin was detected using streptavidin-conjugated horseradish peroxidase (HRP). MotA-S85C, R88C and D121C were strongly labeled by biotin maleimide, MotA-E96C was moderately labeled, but MotA-F29C and G180C were not labeled at all. These results indicate that residues S85, R88, E96 and D121 are exposed at the surface of the MotA structure, whereas residues F29 or G180 may be buried inside the MotA multimeric complex.

### Single particle image analysis of purified MotA

**Figure 5:**
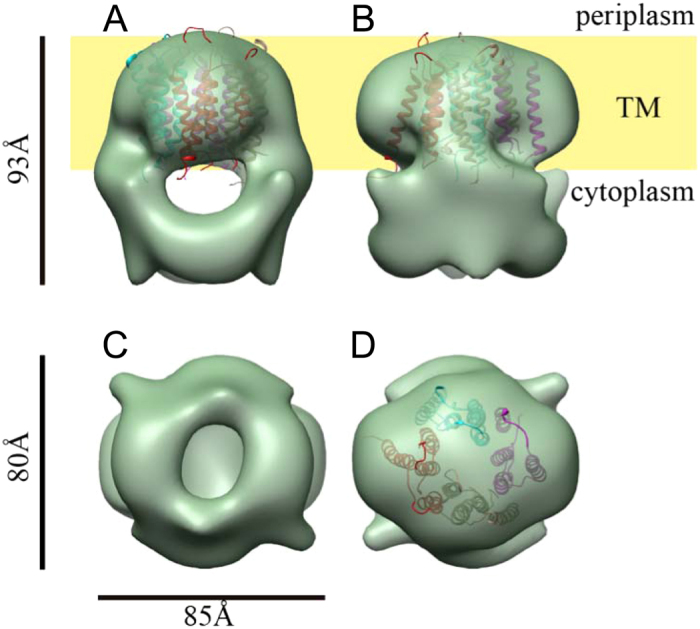
The 3D density map of the MotA multimeric complex by single particle EM image analysis. (**A**) A side view. (**B**) Another side view, with a 90° rotation compared to the one shown in A. The yellow area labeled TM indicates the membrane regions. (**C**) A view from the cytoplasmic side. (**D**) A view from the periplasmic side. An atomic model of the transmembrane region of MotA tetramer complex^13^ was fitted into the transmembrane domain in A,B and D. Four MotA molecules are shown in Cα ribbon representation, each colored in cyan, red, brown and magenta.

The 3D structure of the MotA multimeric complex purified from the 210 kDa fraction was analyzed by single particle EM image analysis. The sample was negatively stained with uranyl acetate, electron micrographs were recorded, and a large number of single particle images were collected and analyzed by 3D image reconstruction, as described in the Methods section (Fig. S4). The stoichiometry of the MotA/MotB complex in *E. coli* has been estimated to be four molecules of MotA and two molecules of MotB^10^, and the 2-fold rotational symmetry of the PomA/PomB complex has been observed by electron tomography^19^. Consequently, 2-fold rotational symmetry was applied for single particle image analysis. The 3D image of the MotA complex was reconstructed at a 25 Å resolution (Fig. 5). The structure of the MotA complex is composed of two parts: a slightly elongated globular part (upper part); and a pair of arch-like domains with spiky projections (lower part). MotA is composed of a four-helix transmembrane domain and a relatively large cytoplasmic domain comprising 144 residues (residues 54–149 and 205–254). The height of the upper globular part is about 50 Å, which is equivalent to the thickness of the lipid bilayer, suggesting that this globular part corresponds to the transmembrane region of MotA. An atomic model of the transmembrane domain, which was generated by molecular dynamics simulation^13^, showed that the MotA tetramer complex fitted well into this region, taking into account that this compartment contains detergent molecules surrounding the transmembrane helices, forming a micelle structure (Fig. 5). The remaining arch-like domains should thus contain the cytoplasmic region of MotA. The total volume of the paired arch-like domains in the 3D map is approximately 84,000 Å^3^, which corresponds to a molecular mass of 68 kDa, assuming a protein density of 1.35 g/cm^3^. This is in good agreement with the molecular mass of 63 kDa calculated for the 576 residues, four times larger than the cytoplasmic 144 residues of MotA. Consequently, the upper and lower parts are likely to correspond to the transmembrane and cytoplasmic domains, respectively, as indicated in Fig. 5.

## Discussion

The stator is one of the most important parts for the proper functioning of the bacterial flagellar motor, and is believed to work as an energy-converting unit that transduces electrochemical potential gradient across the cytoplasmic membrane into mechanical force. The interaction surfaces of the stator and the rotor have been well studied by mutational analyses^4^,^26^,^27^. However, the mechanism governing energy conversion remains unknown because of the lack of structural information on the stator.

Here, we cloned the stator genes from various bacteria and reproduced their expression in the *E. coli* system. Their patterns of expression, observed in the bands in the denaturing gel, were variable, which might be indicative of variations in their molecular sizes and structural stabilities (Fig. S2). Some of them were functional stators within the flagellar motor of *E. coli* or *V. alginolyticus*. A sequence similarity of more than 60% and 90% from native MotA (PomA) and MotB (PomB), respectively, might be required to provide the functional compensation (Table S2).

We overproduced and purified the stator proteins of a thermophilic bacterium, *A. aeolicus*, from the *E. coli* expression system. These stator proteins were chosen because of their observed stabilities. Purified MotA formed multimeric complexes, and is likely to be composed of four MotA molecules. To date, MotA has been thought to form a tetramer through its interaction with the MotB dimer, located at the center of the transmembrane region of the MotA_4_MotB_2_ complex. However, the present study shows that MotA can form a tetramer even in the absence of MotB. Such robust complex formation might be important for the assembly and function of the stator in the motor. A previous study on the *V. alginolyticus* PomA protein showed that PomA formed a dimer instead of a tetramer^11^. This variation might be due to differences in detergents used to solubilize the protein from the membrane and discrepancies in protein stability. In the chemical crosslinking experiment, a strong band corresponding to the dimer was clearly observed, whereas those corresponding to the trimer and tetramer were weak (Fig. 3). It has been thought that four MotA molecules form a pair of dimers within one stator unit. It is possible that the interactions between MotA molecules within the dimer structure are stronger than those between dimers forming the tetrameric unit.

In the experiment aimed at probing surface accessibility, the cytoplasmic amino acid residues (S85C, R88C, E96C, and D121C) were well labeled, whereas the periplasmic residues (F29C and G180C) were not (Fig. 4). In a previous study on the *V. alginolyticus* PomA protein, the periplasmic residues (F29C and G176C), which correspond to F29C and G180C of *A. aeolicus,* respectively, were treated with biotin maleimide in living cells^28^. G176C was labeled, unlike F29C. It was speculated that the F29 residue might be embedded in the pore region of the PomA complex, preventing binding with biotin maleimide. Furthermore, because we used detergent-solubilized MotA in this study, the detergent might have inhibited the reaction between biotin maleimide and the periplasmic residues in the vicinity of the membrane-spanning region. The globular shape of the MotA-complex transmembrane region was visualized by 3D EM image reconstruction (Fig. 5), showing that this region presumably contains a detergent micelle surrounding the transmembrane helices of the four MotA molecules.

Here, we report the first 3D structure of the MotA stator complex formed without MotB (Fig. 5). The shape of this complex is quite different from the corresponding PomA/PomB complex of *V. alginolyticus*, reported previously^19^. Although the resolution is still limited to 25 Å, our present density map clearly shows, for the first time, the shape of the cytoplasmic domain of the stator complex, which is distinctly separated from the transmembrane domain. Because this cytoplasmic domain is solely composed of the cytoplasmic regions of MotA, we measured its volume, estimated the corresponding molecular mass, and found it to be in good agreement with that of the cytoplasmic regions of MotA. An atomic model of the transmembrane domain of the MotA tetramer complex, generated by molecular dynamics simulation^13^, also fitted well into the density map when taking into account the detergent micelle structure surrounding the transmembrane helices (Fig. 5). We are therefore convinced that our 3D density map reflects the actual structure of the MotA tetramer complex.

Until now, various models have been proposed for the molecular mechanism of the stator’s function. Among these is a power-stroke model^29^,^30^,^31^. In this model, the cytoplasmic region of the stator is thought to change its conformation under the action of an ion influx through its channel. Because the MotA complex we purified in this study does not contain MotB, the stator channel was not properly formed, and consequently, the structure of the cytoplasmic domain might have been locked in a single conformation. However, the structure of the cytoplasmic domain should dynamically change under the coupled action of the ion influx through the stator channel and the stator-rotor interaction within the functional flagellar motor of living cells.

## Methods

### Preparation of membrane fractions containing MotA^Aa^

The full-length *motA* gene from *A. aeolicus* (*motA*^*Aa*^) was cloned into a pColdI vector (Takara Bio Inc., Kusatsu, Japan) as previously described^24^. MotA^Aa^ protein was expressed with an N-terminal histidine tag in *E. coli* BL21-CodonPlus(DE3)-RIPL (Agilent Technologies, Santa Clara, CA, USA) cells. The cells were grown in 1.5 L of SB medium [1.2% (w/v) Bacto tryptone, 2.4% (w/v) Bacto yeast extract, 0.5% (w/v) glycerol, 1.25% (w/v) K_2_HPO_4_, 0.38% (w/v) KH_2_PO_4_] containing 100 μg/mL ampicillin, at 37 °C, to an OD_660_ of 0.6–0.8, and 0.5 mM isopropyl-β-d-thiogalactopyranoside (IPTG) was subsequently added to the culture after cooling on ice for 30 min before the culture was prolonged for about 20 h at 16 °C. Cells were collected by centrifugation (7,000 × *g*) and then resuspended in 50 mL of TN buffer [50 mM Tris-HCl (pH 8.0], 200 mM NaCl) containing half a protease inhibitor cocktail tablet (Roche diagnostics) and about 10 mg of lysozyme (Wako, Osaka, Japan). Cells were then disrupted by sonication and ultracentrifuged at 100,000 × *g* for 30 min, and the pellet (membrane fraction) resuspended in TN buffer.

### Solubilization of MotA^Aa^ in detergent

The membrane fraction containing the His-MotA^Aa^ protein was solubilized in 1% (w/v) of CHAPS (Dojindo, Kamimashiki, Japan), Cymal-5 (Anatrace, Maumee, OH, USA), n-dodecyl β-d-maltoside (DDM) (Dojindo), n-decyl β-d-maltoside (DM) (Dojindo), n-dodecylphosphocholine (DPC) (Anatrace), n-octyl β-d-glucoside (OG) (Dojindo), sucrose monocaprate (SMC) (Dojindo), Triton-X100 (Wako) or Tween-20 (Wako) for about 30 min on ice. The detergent solubilized membrane sample was ultra-centrifuged at 100 000 × *g* for 30 min, and separated into pellet (insoluble fraction) and supernatant (soluble fraction). The proteins were resolved by SDS-PAGE and subsequently detected by CBB staining and immunoblot analysis, using the anti-His antibody (MBL, Nagoya, Japan).

### Purification of MotA^Aa^

The membrane fraction containing the His-MotA^Aa^ protein was solubilized by 1% (w/v) Cymal-5 or DM for about 30 min on ice. The detergent-solubilized membrane sample was ultracentrifuged at 100,000 × *g* for 30 min, and the supernatant was mixed with 3 mL of Ni-NTA agarose and then incubated on ice for 1 h. Protein-bound agarose was washed with TN buffer containing 0.1% (w/v) DDM or DM and 50 mM imidazole, and subsequently, proteins were eluted with TN buffer containing 0.1% (w/v) DDM or DM and 500 mM imidazole. The proteins were concentrated using an Amicon Ultra 100 K device (Merck Millipore, Darmstadt, Germany), and His-MotA^Aa^ was purified using gel-filtration chromatography [Superdex 200 Increase 10/300 GL (GE Healthcare, Little Chalfont, UK)] with either TN or PN buffer [50 mM NaPO_4_ (pH 7.0), 150 mM NaCl] containing 0.1% (w/v) DDM or DM.

### Chemical crosslinking

For the reaction with BS^3^ (Thermo Fisher Scientific, Waltham, MA, USA), about 0.5 mg/mL of purified His-MotA^Aa^ protein in PN buffer containing 0.1% (w/v) DM was incubated at 20 °C with 0, 0.2, 0.5, 1, 2 or 5 mM BS^3^, for 5 min. For the reaction with EDC (Thermo Fisher Scientific), about 0.5 mg/mL of purified His-MotA^Aa^ protein in PN buffer containing 0.1% (w/v) DM was incubated at 20 °C with 20 mM EDC for 0, 5, 15, 30, 60 or 120 min. The reactions were quenched by adding 200 mM Tris-HCl (pH 8.0), followed by a 15-min incubation step. The proteins contained in the reaction mixture were separated by SDS-PAGE and detected by CBB staining. The proteins separated by SDS-PAGE were also transferred to PVDF membranes, and immunoblotting was performed using the anti-MotA^Aa^ antibody.

### Introduction of mutations

Point mutations in *motA*^*Aa*^ gene on the plasmid were introduced by performing site-directed mutagenesis (Agilent Technologies). The mutations were confirmed by DNA sequencing.

### Motility assay in soft-agar plates

Motility assays in soft agar plates were performed as previously described^24^. Two microliter of an overnight culture of RP6894 (*E. coli* Δ*motAB*) cells harboring the plasmids pBAD24-*motA*^*Aa*^-A225D (with or without mutations) and pSBETa-*motB*_*2*_^*AE*^, which expressed chimeric MotA/B complex function in *E. coli*^24^, were spotted on TB soft-agar plates [1% (w/v) Bacto tryptone, 0.5% (w/v) NaCl, 0.25% (w/v) Bacto agar] with 0.02% (w/v) arabinose and 0.5 mM IPTG, and were incubated at 30 °C for 26 h.

### Cysteine labeling by biotin maleimide

The membrane fraction of *E. coli* cells expressing the His-MotA^Aa^ protein (with or without Cys substitution mutation) and suspended in TN buffer was solubilized in 1% (w/v) DM, treated with 0.9 mM biotin-maleimide (Sigma-Aldrich, St. Louis, MO, USA), and incubated at 24 °C for 1 h. The biotin maleimide-treated His-MotA^Aa^ was then purified using Ni-NTA agarose, and His-MotA^Aa^ proteins and biotin-labeled His-MotA^Aa^ proteins were checked using SDS-PAGE and CBB staining, followed by blotting analysis using streptavidin-conjugated horseradish peroxidase (HRP) (GE Healthcare).

### Electron microscopy and image analysis

A 3-μL solution of MotA was applied on a glow-discharged continuous carbon grid. The excess solution was removed using a filter paper, and the sample was subsequently stained on the carbon grid with 2% uranyl acetate. Electron microscopy images were recorded with a JEM-3200FSC transmission electron microscope (JEOL, Akishima, Japan) operated at 200 kV and equipped with a F415MP slow-scan CCD camera (TVIPS, Gauting, Germany) at a nominal magnification of 40,000x. About 2,000 particle images were selected manually from 50 electron micrographs and classified into 20 classes using Relion-1.4 (MRC, LMB, Cambridge, UK) and reference-free classification. The best class image in 20 class averages was used as a reference image for automated particle picking, and in total, 54,532 particle images were selected. To reduce the computation time required for analysis, these images were separated into three groups, and reference-free classification was carried out for each group separately. Finally, 5,419 particle images were selected from 150 class averages, and these were used for 3D image reconstruction. The initial structure for the 3D image reconstruction was determined from six class averages by e2initialmodel.py in the EMAN2 package^32^. The final 3D structure was reconstructed using Relion-1.4.

## Additional Information

**Accession code:** The density map of MotA has been deposited in the Electron Microscopy Data Bank, http://www.emdatabank.org/ (accession code: EMD-3417).

**How to cite this article**: Takekawa, N. *et al.* The tetrameric MotA complex as the core of the flagellar motor stator from hyperthermophilic bacterium. *Sci. Rep.* **6**, 31526; doi: 10.1038/srep31526 (2016).

## Supplementary Material

Supplementary Information
