# Supplementary material for: The tetrameric MotA complex as the core of the flagellar motor stator from hyperthermophilic bacterium
Source: Sci Rep. 2016 Aug 17;6:31526. doi: 10.1038/srep31526 (PMC4987623; doi:10.1038/srep31526)
Supplement: Supplementary Information [file srep31526-s1.pdf]

Supplementary information

The tetrameric MotA complex as the core of the flagellar motor stator from hyperthermophilic bacterium

Norihiro Takekawa[a,1], Naoya Terahara[b,1], Takayuki Kato[b,1], Mizuki Gohara[a], Kouta Mayanagi[c,d], Atsushi Hijikata[e], Yasuhiro Onoue[a], Seiji Kojima[a], Tsuyoshi Shirai[e], Keiichi Namba[b,2] and Michio Homma[a,2]

[a]Division of Biological Science, Graduate School of Science, Nagoya University, Chikusa-ku, Nagoya 464-8602, [b]Graduate School of Frontier Biosciences, Osaka University, 1-3 Yamadaoka, Suita, Osaka 565-0871, [c]Medical Institute of Bioregulation, Kyushu University, Higashi-ku, Fukuoka 812-8581, [d]JST, PRESTO, Fukuoka 812-8582, and [e]Department of Bioscience, Nagahama Institute of BioScience and Technology, 1266 Tamura, Nagahama, 526-0829, Japan.

[1]N.Ta., N.Te., and T.K. contributed equally to this work

[2]To whom correspondence should be addressed:

E-mail: g44416a@cc.nagoya-u.ac.jp (M.H.), and keiichi@fbs.osaka-u.ac.jp (K.N.)

Methods

Bacterial strains, plasmids and growth conditions. The bacterial strains and plasmids used in this study are listed in Table S1. The *motAB*^{Aa}, *motB2*^{Aa}, *pomAB*^{Pp}, *pomAB*^{Sb}, *pomAB*^{Sv}, *motAB*^{Tm}, *motAB*^{Va}, *motAB*^{Pp} genes on chromosomal DNA and *pomAB*^{Va} genes on pHFAB were PCR amplified using upstream sense primers and cloned into plasmid vectors, pColdI or pBAD33, as previously described (1). NdeI and XbaI were used for pNT17 and pNT23, NdeI and BamHI were used for pNT18, NdeI and EcoRI were used for pNT19, NdeI and PstI were used for pNT20, pNT21 and pNT22, KpnI and XbaI were used for pNT24, XbaI and PstI were used for pNT25, pNT26, pNT27 and pNT28 as restriction enzyme for cloning. The *E. coli* RP6894 (Δ*motAB*) or *V. alginolyticus* NMB191 (Δ*pomAB*) was transformed by plasmids derived from pBAD33, and the *E. coli* BL21-CodonPlus(DE3)-RIPL was transformed by plasmids derived from pColdI.

Motility assay in soft-agar plate. Motility assay in soft agar plate was performed as described in the article for *E. coli* cells. For *V. alginolyticus* cells, two μl of an overnight culture of cells, expressed stator proteins from other bacteria, in VC medium (0.5% [wt/vol] polypeptone, 0.5% [wt/vol] yeast extract, 0.4% [wt/vol] K_2HPO_4, 3% [wt/vol] NaCl, 0.2% [wt/vol] glucose), were spotted on VPG soft-agar plates (1% [wt/vol] polypeptone, 0.4% [wt/vol] K_2HPO_4, 3% [wt/vol] NaCl, 0.5% [wt/vol] glycerol, 0.25% [wt/vol] Bacto agar) with 0.02% arabinose were incubated at 30ºC for appropriate hours.

References

1. Takekawa N, *et al*. (2015) Sodium-driven energy conversion for flagellar rotation of the earliest divergent hyperthermophilic bacterium. *Sci Rep*

5:12711.

2. Grant SG, Jessee J, Bloom FR, & Hanahan D (1990) Differential plasmid rescue from transgenic mouse DNAs into Escherichia coli methylation-restriction mutants. *Proc Natl Acad Sci USA* 87(12):4645-4649.
3. Parkinson JS & Houts SE (1982) Isolation and behavior of Escherichia coli deletion mutants lacking chemotaxis functions. *J Bacteriol* 151(1):106-113.
4. Okunishi I, Kawagishi I, & Homma M (1996) Cloning and characterization of *motY*, a gene coding for a component of the sodium-driven flagellar motor in *Vibrio alginolyticus*. *J Bacteriol* 178:2409-2415.
5. Yorimitsu T, Sato K, Asai Y, Kawagishi I, & Homma M (1999) Functional interaction between PomA and PomB, the Na^+-driven flagellar motor components of *Vibrio alginolyticus*. *J Bacteriol* 181(16):5103-5106.
6. Guzman LM, Belin D, Carson MJ, & Beckwith J (1995) Tight regulation, modulation, and high-level expression by vectors containing the arabinose pBAD promoter. *J Bacteriol* 177(14):4121-4130.
7. Schenk PM, Baumann S, Mattes R, & Steinbiss HH (1995) Improved high-level expression system for eukaryotic genes in *Escherichia coli* using T7 RNA polymerase and rare ArgtRNAs. *Biotechniques* 19(2):196-198, 200.
8. Fukuoka H, Yakushi T, Kusumoto A, & Homma M (2005) Assembly of motor proteins, PomA and PomB, in the Na^+-driven stator of the flagellar motor. *J Mol Biol* 351(4):707-717.
9. Kojima S, *et al*. (2008) Insights into the stator assembly of the *Vibrio* flagellar motor from the crystal structure of MotY. *Proc Natl Acad Sci USA* 105(22):7696-7701.

10. Asai Y, *et al*. (2003) Ion-coupling determinants of Na^+-driven and H^+-driven flagellar motors. *J Mol Biol* 327(2): 453-463.

Table S1. Strains and plasmids used in this study.

Strain or plasmid	Description	Source or reference
E. coli strains		
DH5α	F⁻λ⁻ *recA1 hsdR17 endA1 supE44 thi-1 relA1 gyrA96*Δ(*argF-lacZYA*) U169 φ80d*lacZ*ΔM15) (Recipient for cloning experiments)	(2)
RP437	wild type for motility	(3)
RP6894	RP437Δ*motAB*	J. S. Parkinson
BL21-CodonPlus (DE3)-RIPL	*E. coli* B F^- *ompT hsdS*(r_B^- m_B^-) dcm^+ Tet^r *gal* λ(DE3) *endA* Hte [*argU proL* Cam^r] [*argU ileY leuW* Strep/$Spec^r$]	Agilent Technology
V. alginolyticus strains		
VIO5	Rif^r Pof^+ Laf^-	(4)
NMB191	VIO5Δ*pomAB*	(5)
Plasmids		
pBAD24	Amp^r P_{BAD}	(6)
pNT7	pBAD24- $motA^{Aa}$	(1)
pSBETa	Km^r P_{T7} argU	(7)
pNT11	pSBETa- $motB_2^{AE}$	(1)
pColdI	Amp^r P_{cspA} (Cold shock expression vector)	Takara
pNT12	pColdI- $motA^{Aa}$	(1)
pNT17	pColdI- $motAB^{Aa}$	This study
pNT18	pColdI- $motB_2^{Aa}$	This study
pNT19	pColdI- $pomAB^{Va}$	This study
pNT20	pColdI- $pomAB^{Pp}$	This study
pNT21	pColdI- $pomAB^{Sb}$	This study
pNT22	pColdI- $pomAB^{Sv}$	This study
pNT23	pColdI- $motAB^{Tm}$	This study
pBAD33	Cm^r P_{BAD}	(6)
pHFAB	pBAD33- $pomAB^{Va}$	(8)
pTF9	pBAD33- *pomApotB*	H. Fukuoka
pNT24	pBAD33- $motAB^{Va}$	This study
pNT25	pBAD33- $pomAB^{Pp}$	This study
pNT26	pBAD33- $motAB^{Pp}$	This study

pNT27	pBAD33- *pomAB*^{Sb}	This study
pNT28	pBAD33- *pomAB*^{Sv}	This study

^{Aa}, genes of *A. aeolicus*; ^{AE}, chimera genes fusing ones of *A. aeolicus* and *E. coli*; ^{Va}, genes of *V. alginolyticus*; ^{Pp}, genes of *P. profundum*; ^{Sb}, genes of *S. benthica*; ^{Sv}, genes of *S. violacea*; ^{Tm}, genes of *T. maritima*; *potB*, a chimeric gene composed of 5'- fragment of *pomB*^{Va} and 3'-fragment of *motB*^{Ec}; Pof^{+}, normal polar flagellar formation; Laf^{-}, defective in lateral flagellar formation; Rif, rifampicin resistant; Amp^{r}, ampicillin resistant; Km^{r}, kanamycin resistant; Cm^{r}, chloramphenicol resistant; P_{BAD}, arabinose promoter; P_{T7}, *T7* promoter; P_{cspA}, promoter of CspA, a major cold shock protein of *E. coli*.

Table S2. Sequence similarity and function of the stator from various bacteria.

		Sequence similarity [*1] against $MotAB^{Ec}$ (%)	Sequence similarity [*1] against $PomAB^{Va}$ (%)	Function in *E. coli*[*2]	Function in *V. alginolyticus*[*3]
$MotAB^{Ec}$	A	-	50	+++	-
	B	-	51		
$PomAB^{Va}$	A	59	-	-	+++
	B	50	-	+ (chimera)[*4]	
$MotAB^{Va}$	A	72	51	+	-
	B	61	51		
$MotAB^{Pp}$	A	75	50	+	nd
	B	62	50		
$PomAB^{Pp}$	A	60	96	-	+++
	B	53	91		
$PomAB^{Sb}$	A	60	91	-	-
	B	53	85		
$PomAB^{Sv}$	A	59	90	-	-
	B	52	74		
$MotAB^{Tm}$	A	62	74	nd	nd
	B	55	52		
$MotAB^{Aa}$	A	61	73	+ (chimera)[*5]	nd
	B_1	57	64		
	B_2	57	60		

$MotAB^{Ec}$, proton-driven stator proteins of *E. coli*; $PomAB^{Va}$, sodium-driven stator proteins of polar flagellum of *V. alginolyticus*; $MotAB^{Va}$, proton-driven stator proteins of lateral flagella of *V. alginolyticus*; $MotAB^{Pp}$, putative proton-driven stator proteins of *P. profundum*; $PomAB^{Pp}$, putative sodium-driven stator proteins of *P. profundum*; $PomAB^{Sb}$, putative sodium-driven stator proteins of *S. benthica*; $PomAB^{Sv}$, putative sodium-driven stator proteins of *S. violacea*; $MotAB^{Tm}$, putative sodium-driven stator proteins of *T. maritima*; $MotAB^{Aa}$, sodium-driven stator

proteins of *A. aeolicus*. [*1], Amino acid sequences of each proteins were align and sequence similarity was calculated. [*2], Various stator proteins were expressed in *E. coli* Δ*motAB* cells and motility of the cells was observed. [*3], Various stator proteins were expressed in *V. alginolyticus* VIO5 Δ*pomAB* (which formed polar flagellum but not lateral flagella) cells and motility of the cells was observed. +++, similar motility to wild type cells; +, motile but significantly reduced. [*4], native PomAB^{Va} did not function in *E. coli* but chimeric PomAPotB, the periplasmic region of PomB was replaced with MotB of *E. coli*, did function as previously described (10). [*5], native MotAB^{Aa} did not function in *E. coli* but chimeric MotAB^{AE}, the periplasmic region of MotB was replaced with one of *E. coli*, did function as previously described (1).

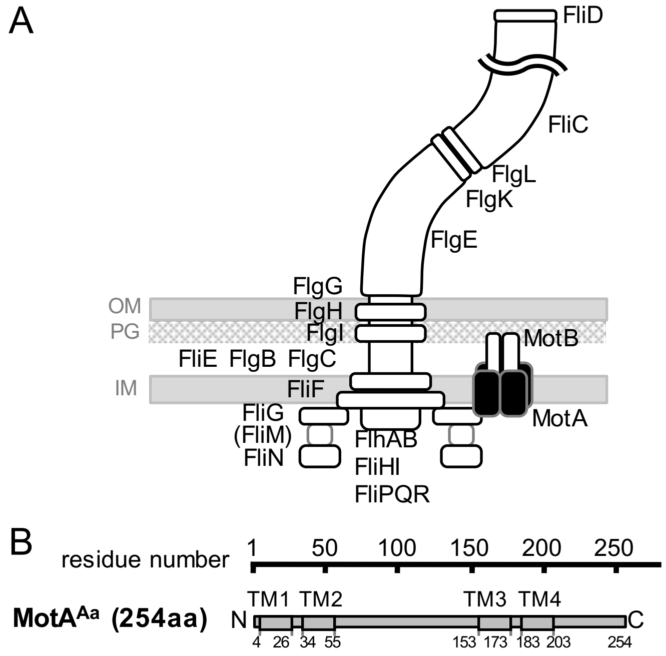

Fig. S1. Schematic cartoon of the flagellum and the stator proteins. (A) The flagellum is a large complex composed of many proteins and consists of a filament, a hook and a basal body. *A. aeolicus* has most genes for flagellar component except for FliM. (B) Schematics of primary structures of *A. aeolicus* MotA. MotA is a four TM protein. OM, outer membrane; PG, peptidoglycan layer; IM, inner membrane.

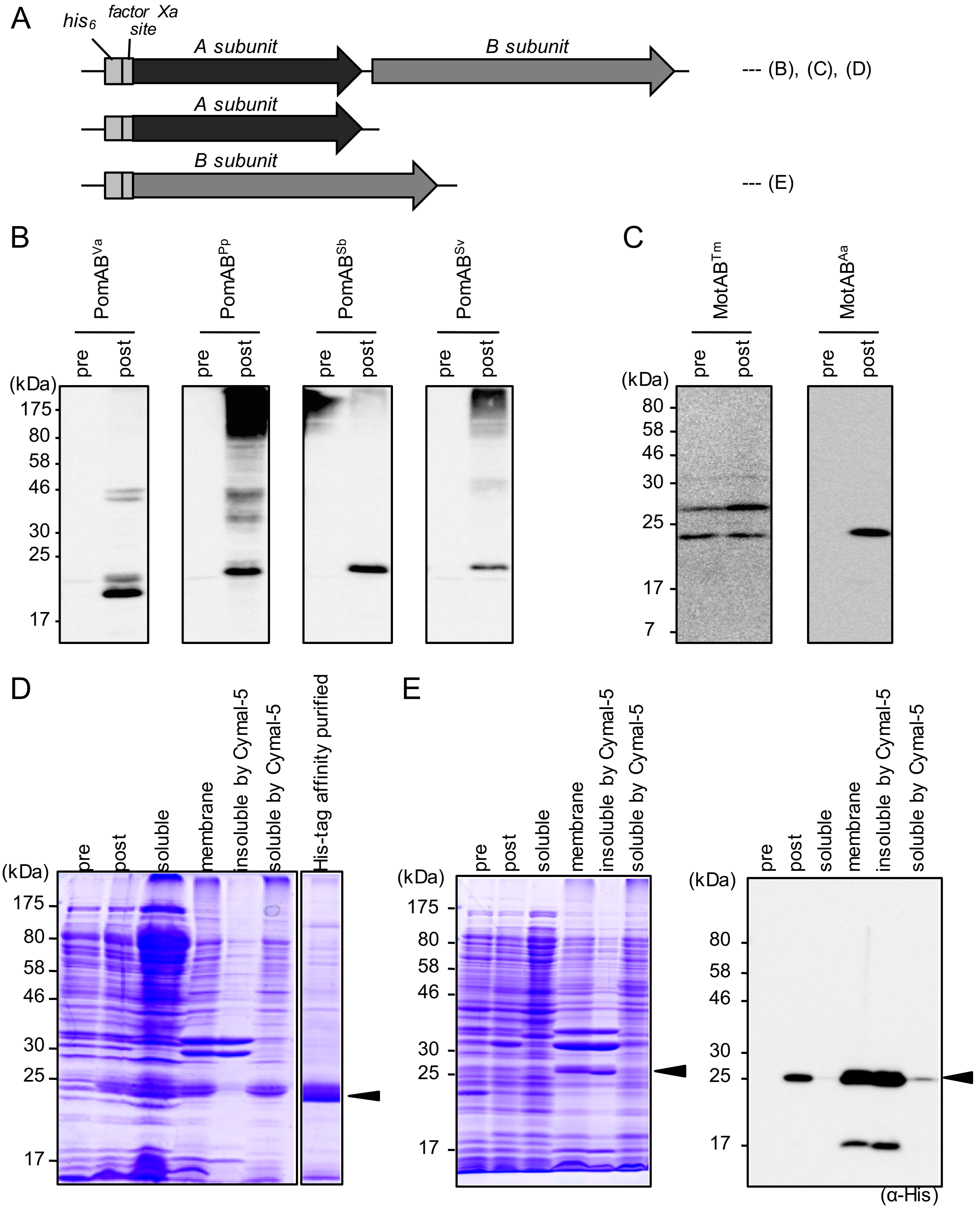

Fig. S2. Expression of the stator proteins from various bacteria. (A) Schematic of the cloning. The *pomA/pomB genes from V. alginolyticus* (Va), *P. profundum* (Pp), *S. benthica* (Sb) and *S. violacea* (Sv), and *motA/motB* genes from *T. maritima* (Tm), and *A. aeolicus* (Aa) were cloned into plasmid vector pColdI for (B), (C) and (D). The *motB*$_2$ genes from *A. aeolicus* were cloned into pColdI for (E). (B, C) *E. coli* cells transformed by plasmid were cultured and pre-induction (pre) and post-induction (post) whole cell lysate samples were analyzed by SDS-PAGE and immunoblotting using anti-His antibody. (D, E) *E. coli cells* transformed by plasmid were cultured and pre-induction (pre) and post-induction (post) whole cell lysate samples, supernatant (soluble) and precipitate (membrane) after sonication and ultra-centrifugation, the samples insoluble and soluble by Cymal-5, which were precipitate and supernatant, respectively, after treatment with 1% Cymal-5 and ultra-centrifugation, and His-tag affinity purified sample were analyzed by SDS-PAGE, CBB staining and immunoblotting using anti-His antibody. Black arrowhead, His-MotA^{Aa} (D) or His-MotB$_2^{Aa}$ (E).

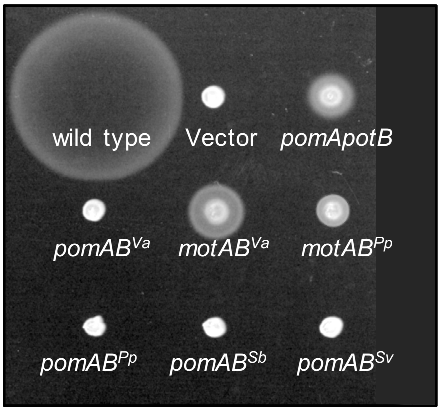

Fig. S3. Function of the stator proteins from various bacteria in *E. coli*. Motility assay in soft-agar plate of *E. coli* cells producing stators from various bacteria was performed. Overnight cultures were spotted on TB-0.25% agar plate containing 0.02% arabinose and incubated at 30° C for 6 h. *pomApotB*, chimeric stator of *Vibrio alginolyticus.* See also Table S2.

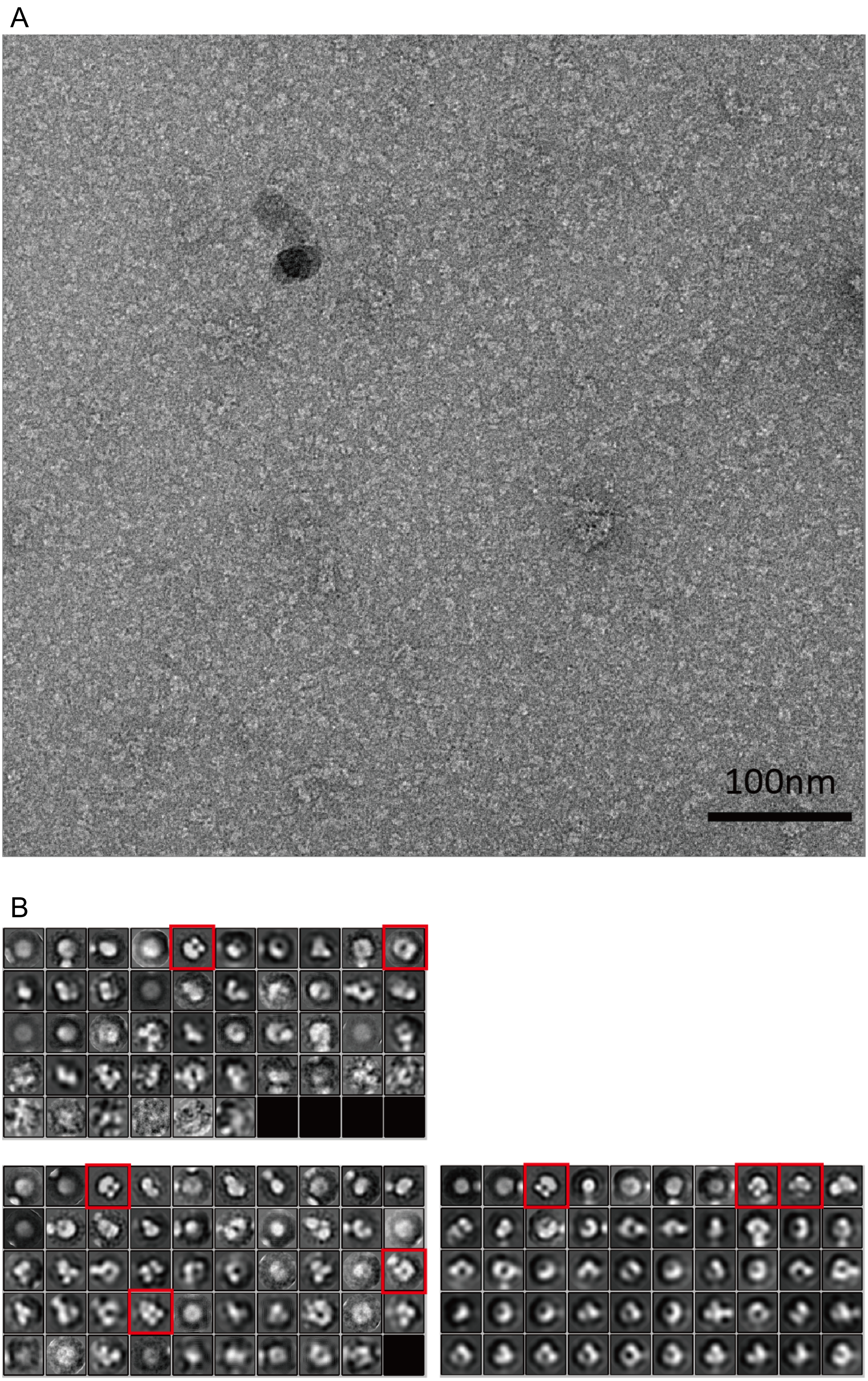

Fig. S4. Electron micrograph of the MotA complex particles and 2D class averages. (A) Typical electron micrograph of the negatively stained MotA complex. (B) Results of 2D classification of the three groups of particle images. Particle images contributed to the class averages indicated by red box were used for 3D image reconstruction.
